# Industrial development alters wolf spatial distribution mediated by prey availability

**DOI:** 10.1002/ece3.10224

**Published:** 2023-06-28

**Authors:** Hannah Boczulak, Nicole P. Boucher, Andrew Ladle, Mark S. Boyce, Jason T. Fisher

**Affiliations:** ^1^ School of Environmental Studies University of Victoria Victoria British Columbia Canada; ^2^ Department of Biological Sciences University of Alberta Edmonton Alberta Canada

**Keywords:** anthropogenic disturbance, camera traps, elk, human activity, mule deer, predator–prey dynamics, prey availability, wolves

## Abstract

Increasing resource extraction and human activity are reshaping species' spatial distributions in human‐altered landscape and consequently shaping the dynamics of interspecific interactions, such as between predators and prey. To evaluate the effects of industrial features and human activity on the occurrence of wolves (*Canis lupus*), we used wildlife detection data collected in 2014 from an array of 122 remote wildlife camera traps in Alberta's Rocky Mountains and foothills near Hinton, Canada. Using generalized linear models, we compared the occurrence frequency of wolves at camera sites to natural land cover, industrial disturbance (forestry and oil/gas exploration), human activity (motorized and non‐motorized), and prey availability (moose, *Alces alces*; elk, *Cervus elaphus*; mule deer, *Odocoileus hemionus*; and white‐tailed deer, *Odocoileus virginianus*). Industrial block features (well sites and cutblocks) and prey (elk or mule deer) availability interacted to influence wolf occurrence, but models including motorized and non‐motorized human activity were not strongly supported. Wolves occurred infrequently at sites with high densities of well sites and cutblocks, except when elk or mule deer were frequently detected. Our results suggest that wolves risk using industrial block features when prey occur frequently to increase predation opportunities, but otherwise avoid them due to risk of human encounters. Effective management of wolves in anthropogenically altered landscapes thus requires the simultaneous consideration of industrial block features and populations of elk and mule deer.

## Graphical abstract

To evaluate the effects of industrial features and human activity on the occurrence of wolves (*Canis lupus*), we used wildlife detection data from an array of 122 remote wildlife camera traps in Alberta's Rocky Mountains and foothills near Hinton, Canada, 2014. Industrial block features (well sites and cutblocks) and prey (elk and mule deer) availability interacted to influence wolf occurrence, but models including motorized and non‐motorized human activity were not strongly supported. Our results suggest that wolves risk using industrial block features when prey occur frequently to increase predation opportunities, but otherwise avoid them due to perceived risk of human encounters, regardless of human activity.

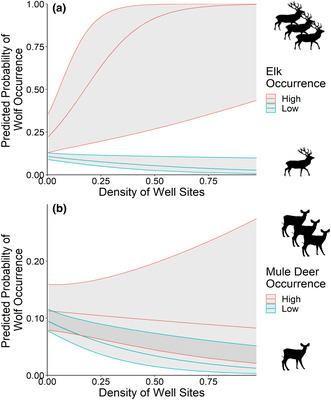

## 1 INTRODUCTION

Anthropogenic landscape modifications and human activity play a significant role in reshaping the spatial distributions of wildlife and the dynamics of interspecific interactions such as predation (Fisher & Burton, 2018; Fisher & Ladle, 2022). Human disturbance alters the quality, quantity, and spatial configuration of habitats, which influences species' behaviors, distributions, and populations (Ciuti et al., 2012; Newbold et al., 2015). The differential ability of species to adjust to these novel habitats leads to winners and losers—those that benefit from human disturbance and those that do not—in the altered landscape, possibly destabilizing ecological interactions between species (Fisher & Burton, 2018). Consequently, spatial co‐occurrence between interacting species is likely to be affected by these changes as they navigate through human‐modified landscapes with large‐scale implications for wildlife populations and biodiversity (Muhly et al., 2011; Shackelford et al., 2018).

In the Nearctic, the western boreal forest ecosystem continues to face increasing anthropogenic development, resulting in landscapes being fragmented by industrial features from resource extraction (forestry, oil and gas exploration, and coal mining) (Pickell et al., 2015; Venier et al., 2014). These industrial features include both linear features—trails, seismic lines, roads, and pipelines—as well as polygonal block features—cutblocks and well sites—that together drastically modify the landscape, resulting in significant habitat loss and fragmentation (Dabros et al., 2018; Pickell et al., 2015). Simultaneously, human activity has increased within the landscape, as linear features increase access to previously remote areas. These human disturbances are of major concern for wildlife conservation because they have the potential to broadly affect biotic communities through changes in the behavior, distribution, and abundance of interacting species (Hebblewhite, 2017).

Industrial habitat alteration and human activity influence predator–prey dynamics by altering predation risk and resource availability across a landscape (Boucher et al., 2022; Dickie et al., 2017; Whittington et al., 2019). Wolves (*Canis lupus*) and other large predators often show avoidance of areas with high human activity due to the risk of interactions with humans (Muhly et al., 2011; Rogala et al., 2011; Whittington et al., 2019). Ungulate prey indirectly benefit by using areas with high human density as a refuge from predators (Berger, 2007; Muhly et al., 2011). However, predators also can benefit from human‐induced landscape change by selecting and disproportionately using linear features to increase their movement rates and search efficiency while hunting (Boucher et al., 2022; DeMars & Boutin, 2018; Dickie et al., 2017). Furthermore, altered prey availability—which we define here as spatial co‐occurrence, as proximity is a requirement of availability—can benefit predators like wolves in disturbed landscapes (DeCesare et al., 2010; Holt, 1977). Following human developments, early seral forests become more abundant and support higher densities of primary prey species, including moose (*Alces alces*), elk (*Cervus elaphus*), mule deer (*Odocoileus hemionus*), and white‐tailed deer (*O. virginianus*) (Latham, Latham, McCutchen, et al., 2011). These prey species supplement the diets of wolves, bolstering wolf population densities, and leading to increased predation on vulnerable species such as the threatened Boreal woodland caribou (*Rangifer tarandus caribou*) (Latham, Latham, McCutchen, et al., 2011).

Thus, spatial occurrence of wolves within an industry‐managed landscape is likely modified by the interactive effects of anthropogenic features, human activity, and prey occurrence. To evaluate these potentially interactive effects on wolves, we used mammal occurrence data collected in 2014 from an array of 122 wildlife camera traps in the eastern slopes beside Jasper National Park and the adjacent area along the eastern Rocky Mountain foothills of Alberta, Canada. This region supports a diverse large mammal community and abundant wolf population (Webb et al., 2009) but also has undergone significant resource extraction from energy (oil and gas) exploration and forestry. Using generalized linear models, we weighed evidence for competing hypotheses that industrial features, human recreation (motorized and non‐motorized), and prey availability alter the frequency of wolf occurrence on the landscape. First, we hypothesized that wolves would occur more frequently on industrial features due to the improved hunting efficiency conferred by these features, such that models including both natural and industrial features would better explain wolf occurrence than natural features alone. Second, we expected increased wolf occurrence on industrial features when ungulate prey occurrence was high due to increased predation opportunities outweighing risk from humans, predicting that models with an interaction term between industrial features and ungulate availability would better explain wolf occurrence frequency than industrial features or ungulate availability alone. Lastly, we expected less frequent wolf occurrences in areas with high human activity with models including non‐motorized and motorized activity explaining wolf occurrence better than natural features alone.

## 2 METHODS

### 2.1 Study area

**FIGURE 1:**
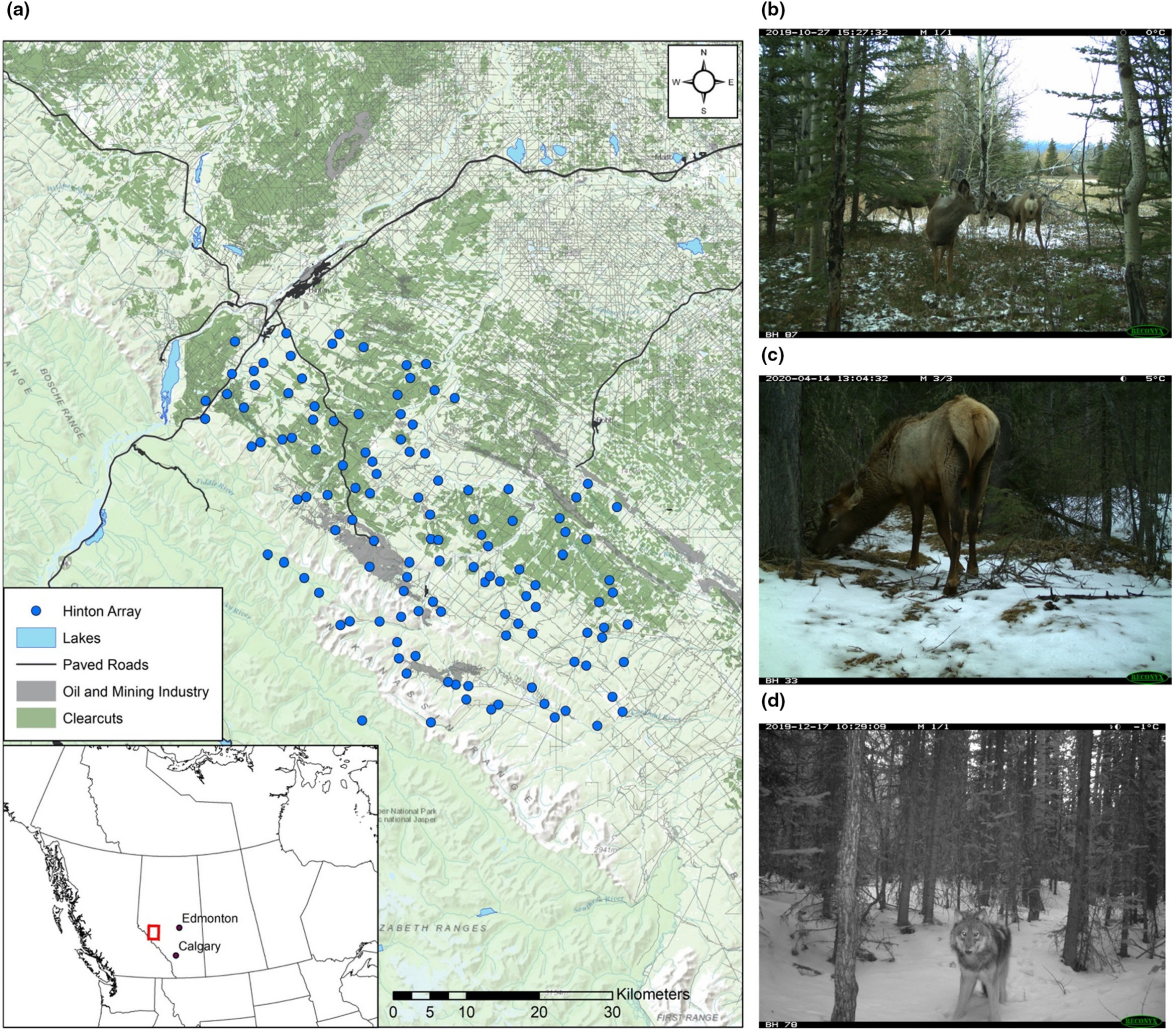
(a) Map of the study area in Alberta's Rocky Mountains and foothills near Hinton, including camera locations (blue circles), major waterbodies (blue polygons), major paved roads (black lines), and industrial disturbance features (clearcuts = green, oil and mining industry = gray polygons and lines). (b) mule deer, *Odocoileus hemionus*. (c) Elk, *Cervus canadensis*. (d) gray wolf, *Canis lupus*.

Our study area encompasses part of central Alberta's Rocky Mountains and foothills regions (Figure 1a), which support an abundant wolf population (Webb et al., 2009), diverse community of ungulates (Figure 1b–d), and significant resource extraction. Wolf harvest is highly prevalent in this region, and the leading causes of mortality for adult wolves are trapping followed by hunting (Robichaud & Boyce, 2010; Webb et al., 2011). This region includes protected areas where recreational motorized off‐road vehicles are prohibited—including Jasper National Park and Whitehorse Wildland Park—and public lands where motorized activity has limited restrictions and is dominant on the landscape. An increasingly heavy presence of industrial disturbance from oil and gas extraction, open‐pit coal mining, and timber harvest is present in the unprotected areas of this landscape. These industries contribute to a high density of linear features, including paved and unpaved roads, trails, and conventional (5–10 m) and low‐impact seismic lines (2–5 m), and their anthropogenic footprint is growing rapidly (Linke & McDermid, 2012). As well, polygonal features from industrial activities are spread across the landscape, including cutblocks and well sites. Periodic wildfires have occurred in the region in previous decades, but with heavy fire suppression. The study area consists of high‐elevation mountainous terrain in the west, with lower elevations in the foothills to the east. Forest cover consists of fir (*Abies* spp.), aspen (*Populus tremuloides*), balsam poplar (*P. balsamifera*), lodgepole pine (*Pinus contorta*), and spruce (*Picea* spp.).

### 2.2 Camera trap sampling

We used wildlife detection data from 122 Reconyx HC500 cameras, which were active across the study area from May 1 to November 1, 2014, as part of a 3‐year project conducted by Ladle et al. (2017). We chose to only include 1 year of data in our study as other years in this project utilized camera site rotation (see Ladle et al. (2017) for further details on camera deployment). Additionally, we aimed to limit temporal variation within our analysis by restricting the study to a single season. Cameras were deployed on human‐use trails (i.e., linear features created by humans, excluding active roads). Each camera site was deployed in a 50‐km^2^ cell using a systematic design and ensuring sites were evenly distributed spatially on trails across the landscape (Ladle et al., 2017). The systematic design allows for inference across the study area, defined as the minimum convex polygon around the camera sites, and for results to be generalized across the rest of Alberta's foothills. A minimum distance of 1‐km was maintained between camera locations to ensure independence and reduce spatial autocorrelation (median distance between cameras: 24.6 km). We placed cameras obliquely to the trail, approximately 1–3 m away, to ensure fast‐moving off‐highway vehicles (OHV) were detected. Cameras were active 24 h a day, set to “high sensitivity” and captured 3–5 pictures in rapid succession with no delay between photos when triggered. No bait or lure was used. Every 20–40 days, we downloaded photos from the cameras and replaced their batteries.

We classified images by date, time, and presence of wolf, white‐tailed deer, mule deer, elk, moose, non‐target species, and human activity. If human recreation was present, we identified the type of recreation and categorized the event as either motorized (quad, vehicle, motorbike, snowmobile) or non‐motorized (biker, hiker, horse rider, snowshoer, runner). Images of staff members were removed from the data. From these data, we determined the weekly occurrence of each species, using a binomial response (1 = detection; 0 = non‐detection). Camera weeks with <7 days of active collection were removed to ensure accurate measures of detectability.

### 2.3 Covariates

For each camera site, we extracted variables describing natural land cover, industrial features, prey availability, and human activity (Table 1). Natural land cover was measured as the proportion of each habitat type within a 1000‐m buffer centered around each camera site, using Alberta Vegetation Inventory (AVI) spatial layers (Government of Alberta). Elevation (m) was extracted from each camera location using a digital elevation model. Anthropogenic disturbance features were derived from the Alberta Biodiversity Monitoring Institute (2014) Wall‐to‐Wall Human Footprint Inventory and categorized into block features (cutblocks, and active and abandoned well sites) and linear features (seismic lines, roads, pipelines, and trails). For each camera site, we determined the density of well sites, cutblocks, roads, and trails within a 1000‐m buffer, as well as distance to the nearest well site and road (Table 1).

**TABLE 1:** Descriptions of natural land cover and industrial disturbance covariates, with the mean ± standard error (SE) for all camera sites.

Covariate	Description	Mean ± SE	Range (Min–Max)
Natural land cover			
Dense Conifer Forest	>70% crown closure; >80% coniferous forest	21.88 ± 0.54	0.17–81.05
Moderate Conifer Forest	31%–69% crown closure; >80% coniferous forest	33.59 ± 0.71	1.13–90.42
Open Conifer Forest	<30% crown closure; >80% coniferous forest	3.10 ± 0.09	0.00–12.40
Mixed Wood Forest	21%–79% coniferous forest	7.76 ± 0.42	0.00–63.15
Broadleaf Forest	<20% coniferous forest	0.91 ± 0.08	0.00–13.33
Treed Wetland	>6% crown closure; “wet” or “aquatic” moisture regime	0.65 ± 0.08	0.00–18.40
Open Wetland	<6% crown closure; “wet” or “aquatic” moisture regime	0.11 ± 0.02	0.00–6.56
Shrubs	>25% shrub cover; <6% tree cover; “dry” or “mesic” moisture regime	8.31 ± 0.32	0.00–45.55
Herbaceous	<25% shrub cover; <6% tree cover; “dry” or “mesic” moisture regime	3.65 ± 0.18	0.00–28.74
Barren Land	<6% vegetation cover	5.69 ± 0.40	0.00–60.33
Water	Waterbodies (e.g., lakes and ponds) with >6% standing or flowing water (e.g., rivers)	0.11 ± 0.06	0.00–3.02
Industrial features			
Block features			
Well site density	Density of active and abandoned well sites	0.08 ± 0.01	0.00–1.06
Well site distance	Distance to the nearest active and abandoned well sites	4.43 ± 0.15 km	0.35–23.14 km
Cutblock density	Density of cutblocks	17.14 ± 0.88	0.00–89.73
Linear features			
Road density	Unimproved, gravel, and paved roads	0.47 ± 0.02	0.00–1.76
Trail density	Off‐highway vehicle trails, conventional and low‐impact seismic lines	2.10 ± 0.04	0.00–7.41
Road Distance	Distance to the nearest road (Unimproved, gravel, or paved)	1.32 ± 0.31 km	0.00–12.04 km

*Note*: Covariates were extracted as proportions within a 1000‐m buffer surrounding camera sites, unless otherwise specified as a measure of density within the 1000‐m buffer or distance of the nearest feature to the camera site (km).

Measures of prey availability (elk, white‐tailed deer, mule deer, and moose) and human activity (motorized and non‐motorized) were derived from the weekly camera detection dataset and were represented by a proportion of the number of weeks the focal species or human activity was detected to the total number of active camera sampling weeks, as a measure of their spatial distributions and intensity of site use. This measure of occurrence frequency accounts for variation in sampling effort between sites (e.g., camera failures) and detection rate outliers (e.g., groups of individuals causing repeated detections) (Fisher & Ladle, 2022). We assumed that zero observations of target species or human activity were true zeros indicating frequency of space use, rather than an error in detections or cameras (Stewart et al., 2018). While other ungulates exist in the study area, we only included elk, white‐tailed deer, mule deer, and moose within the analysis because other prey species (e.g., bighorn sheep, *Ovis canadensis*) had limited detections.

### 2.4 Statistical analysis

Proportional binomial generalized linear models (binomial errors, logit link), fit using the function “glm” from the *stats* package in R version 4.0.2 (R Core Team, 2022), were used to estimate the predicted response of wolf occurrence frequency in relation to natural and anthropogenic features, prey availability, human activity, and interactions between these variables. The response variable was a single metric from each site: proportion of the number of weeks with wolf detections to the number of weeks without wolf detections. Prior to inclusion within models, we scaled all explanatory variables (mean = 0, standard deviation = 1) and checked for collinearity using Pearson correlation coefficients with a threshold of 0.7. If this threshold was exceeded, we either combined related variables or if dissimilar, ran separate models for each variable.

We chose first to partition out variance due to natural heterogeneity by modeling wolf occurrence frequency against natural land cover in a core model. The core model originally included all non‐collinear natural land cover variables (Table 1) and was reduced with backward stepwise model selection based on Akaike's information criterion (AIC), using the “stepAIC” function in the R package *MASS* (Ripley et al., 2013). This process created a core model that only included the natural land cover features that best explained wolf occurrence frequency.

Next, we created a series of 22 candidate models using variables for industrial development, human recreation, and/or prey species occurrence to weigh evidence for the corresponding hypotheses (Table 2). We included the core model in all candidate models to control for habitat variables that were not of interest in our hypotheses. We calculated AIC for each model to identify which best explained wolf occurrence frequency, with the most supported model having the lowest ∆AIC score and highest AIC weight (AIC_w_) (Burnham & Anderson, 2002). We considered any candidate models with ∆AIC < 2 to be well supported and used these models to make inferences. To validate models, we used *k*‐fold cross‐validation to determine the adjusted delta, which represents the bias‐corrected average mean‐squared error for the model (Canty, 2002; Davison & Hinkley, 1997; Roberts et al., 2017). For the most supported model(s), we produced predicted probability plots and represented high‐ and low‐occurrence frequencies using the 95th and 5th percentiles, respectively.

**TABLE 2:** Hypotheses explaining the drivers of wolf (*Canis lupus*) occurrence frequency in central Alberta's Rocky Mountains and foothills regions in 2014, with the corresponding candidate models, model structure, Akaike's Information Criterion (AIC) values, ∆AIC scores, and AIC weights for each model.

Wolf occurrence is influenced by…	Model number	Variables	Rank	AIC	∆AIC	AIC weight	*k*‐Fold adjusted delta
Natural land cover	CM	NATURAL		528.1			
Industrial features	1	LINEAR	11	522.78	18.50	0.00	0.025
	2	BLOCK	6	512.12	7.84	0.01	0.022
	3	LINEAR + BLOCK	5	511.87	7.59	0.01	0.023
Prey species occurrence	4	WHITE‐TAILED DEER	18	529.05	24.77	0.00	0.026
	5	MULE DEER	21	529.64	25.36	0.00	0.026
	6	MOOSE	20	529.42	25.14	0.00	0.026
	7	ELK	22	529.93	25.65	0.00	0.026
Industrial features mediated by prey species occurrence	8	WHITE‐TAILED DEER + LINEAR + WTD*LINEAR	16	525.18	20.90	0.00	0.028
	9	WHITE‐TAILED DEER + BLOCK + WTD*BLOCK	4	510.32	6.05	0.03	0.024
	10	MULE DEER + LINEAR + MD*LINEAR	3	509.45	5.17	0.04	0.025
	11	MULE DEER + BLOCK + MD*BLOCK	2	505.02	0.74	0.37	0.022
	12	MOOSE + LINEAR + MOOSE*LINEAR	19	529.25	24.97	0.00	0.027
	13	MOOSE + BLOCK + MOOSE*BLOCK	8	516.35	12.08	0.00	0.023
	14	ELK + LINEAR + ELK*LINEAR	7	514.37	10.09	0.00	0.027
	15	ELK + BLOCK + ELK*BLOCK	1	504.28	0.00	0.54	0.022
Human activity	16	MOTORIZED	17	527.64	23.37	0.00	0.026
	17	NON‐MOTORIZED	9	521.19	16.91	0.00	0.026
	18	MOTORIZED + NON‐MOTORIZED	10	522.19	17.91	0.00	0.026
Prey species occurrence mediated by human activity	19	WHITE‐TAILED DEER + MOTORIZED + NON‐MOTORIZED	12	523.95	19.67	0.00	0.027
	20	MULE DEER + MOTORIZED + NON‐MOTORIZED	15	524.19	19.91	0.00	0.027
	21	MOOSE + MOTORIZED + NON‐MOTORIZED	13	524.13	19.85	0.00	0.026
	22	ELK + MOTORIZED + NON‐MOTORIZED	14	524.17	19.89	0.00	0.027

*Note*: The core model (CM) was included in every candidate model to account for baseline occurrence of wolves in relation to natural land cover. NATURAL = Proportion of natural landscape features; LINEAR = Density of linear features; BLOCK = proportion of block features (well sites and cutblocks); WHITE‐TAILED DEER or WTD = White‐tailed deer (*Odocoileus virginianus*) occurrence frequency; MULE DEER or MD = Mule deer (*O. hemionus*) occurrence frequency; MOOSE = moose (*Alces alces*) occurrence frequency; ELK = Elk (*Cervus canadensis*) occurrence frequency; MOTORIZED = Motorized human activity (quad, vehicle, motorbike, snowmobile) occurrence frequency; NON‐MOTORIZED = Non‐motorized (biker, hiker, horse rider, snowshoer, runner) occurrence frequency. * denotes an interaction between variables.

## 3 RESULTS

### 3.1 Camera trap sampling

**FIGURE 2:**
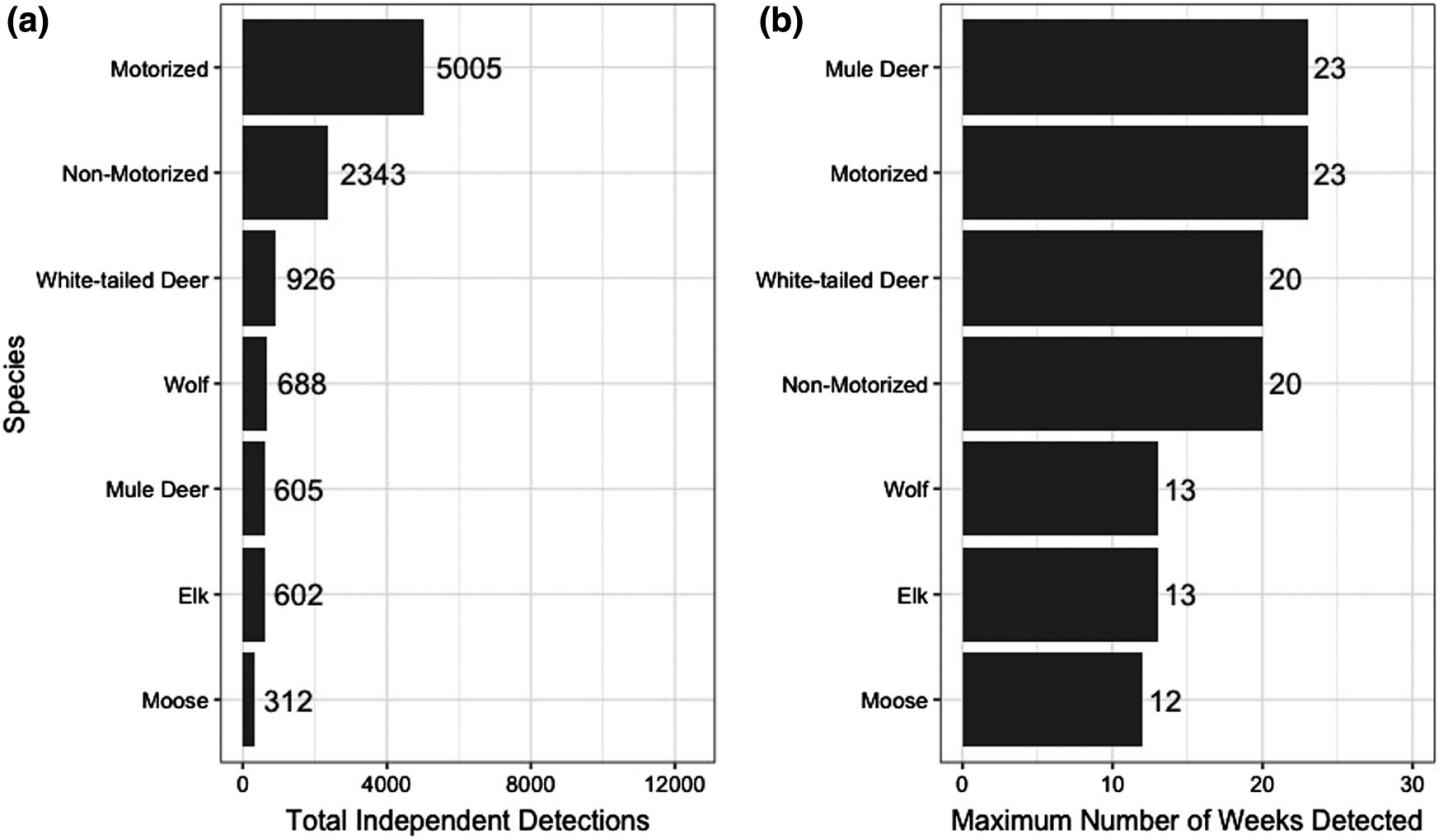
(a) Total independent detections and (b) maximum number of weeks detected for wolves (*Canis lupus*), white‐tailed deer (*Odocoileus virginianus*), mule deer (*O. hemionus*), moose (*Alces alces*), elk (*Cervus canadensis*), and human activity (motorized = quad, vehicle, motorbike, or snowmobile; non‐motorized = biker, hiker, horse rider, snowshoer, or runner), based on images captured by camera traps in the Hinton study area in Alberta's Rocky Mountains and Foothills, 2014.

**FIGURE 3:**
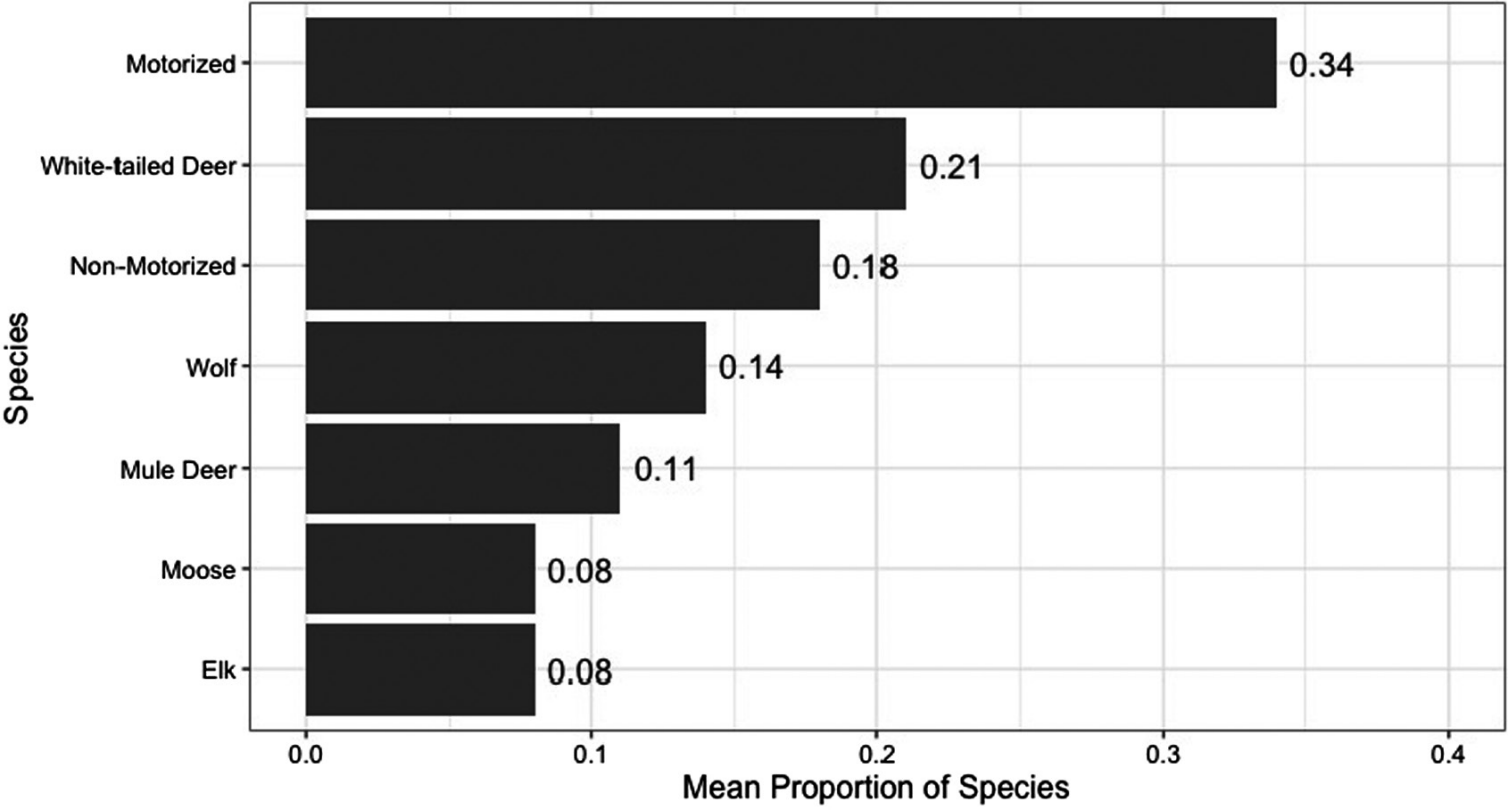
Mean occurrence frequency (number of weeks the focal species was present divided by the total active camera trap sampling weeks) of wolves (*Canis lupus*), white‐tailed deer (*Odocoileus virginianus*), mule deer (*O. hemionus*), moose (*Alces alces*), elk (*Cervus canadensis*), and human activity (motorized = quad, vehicle, motorbike, or snowmobile; non‐motorized = biker, hiker, horse rider, snowshoer, or runner), based on images captured by camera traps in the Hinton study area in Alberta's Rocky Mountains and Foothills, 2014.

Camera sites were sampled for a mean of 18 weeks (range: 1–25 weeks). Across all cameras, we recorded 5005 observations of motorized activity, 2343 of non‐motorized, 926 of white‐tailed deer, 688 of wolves, 605 of mule deer, 602 of elk, and 312 of moose (Figure 2). The highest proportion of weekly detections were motorized activity (0.34), followed by white‐tailed deer (0.21), non‐motorized activity (0.18), wolves (0.14), mule deer (0.11), moose (0.08), and elk (0.08) (Figure 3). Wolves co‐occurred with white‐tailed deer (*n* = 76 camera sites), mule deer (*n* = 53), moose (*n* = 53), and elk (*n* = 27). We identified no collinearity between independent predictors, except for abandoned and active well pads (*r* = .71) which we added together into one measure.

### 3.2 Model selection

**FIGURE 4:**
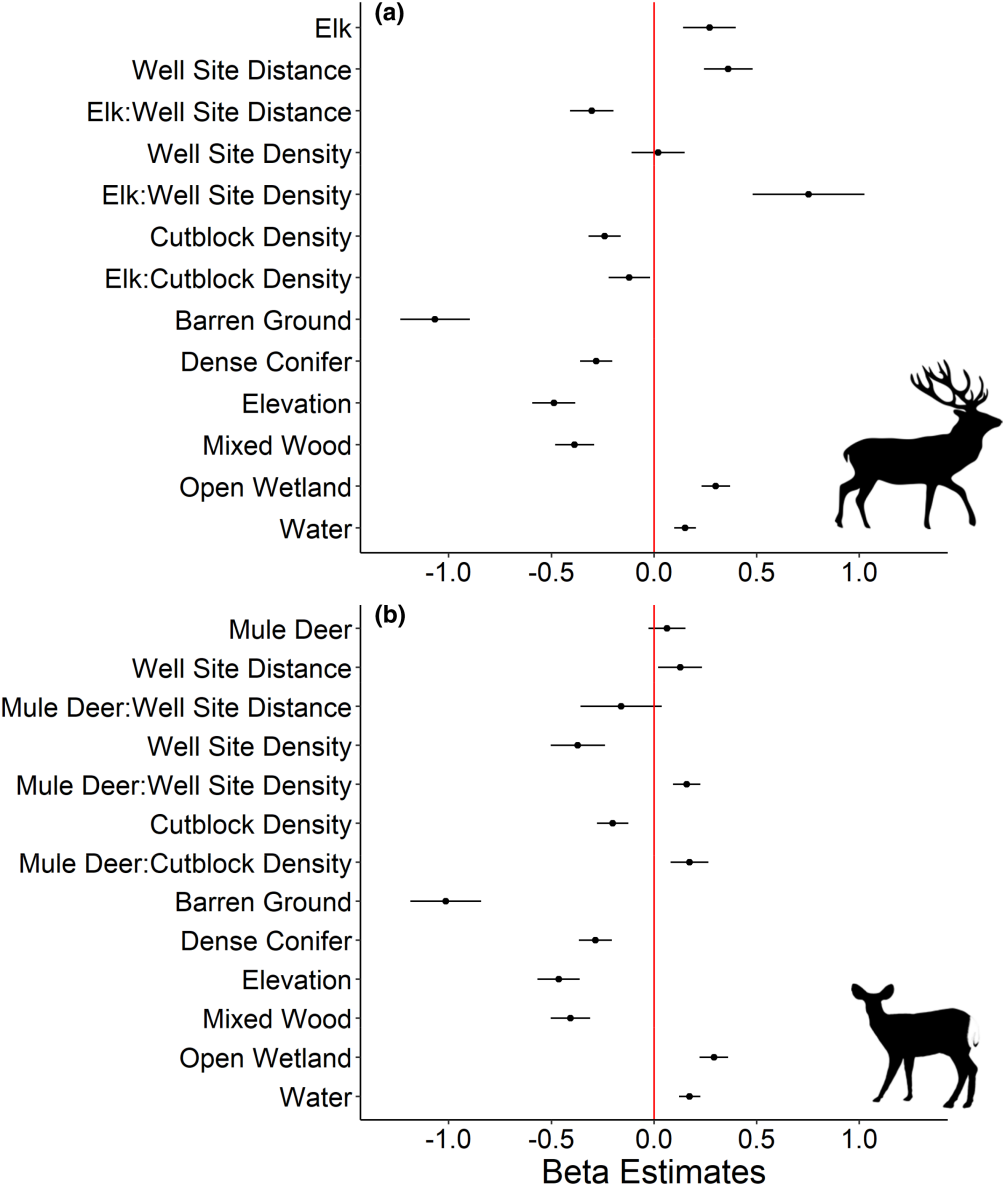
Beta coefficient estimates from the two most supported models relating wolf (*Canis lupus*) occurrence frequency to industrial block features, natural land cover, and prey occurrence frequencies of (a) elk (*Cervus canadensis*) and (b) mule deer (*Odocoileus hemionus*) at camera site locations in central Alberta's Rocky Mountains and foothills regions in 2014.

Of the 22 candidate models explaining wolf occurrence frequency, the two most supported models described the hypothesis that wolves will occur more frequently at higher densities of industrial block features when ungulate prey are relatively abundant (Table 2). These two best‐supported models included variables describing block features and the occurrence frequency of either elk (AIC_w_ = 0.54) or mule deer (AIC_w_ = 0.37; Table 2 and Figure 4). Models including motorized or non‐motorized human activity covariates were not strongly supported (Table 2).

### 3.3 Wolf occurrence relative to elk and block features

**FIGURE 5:**
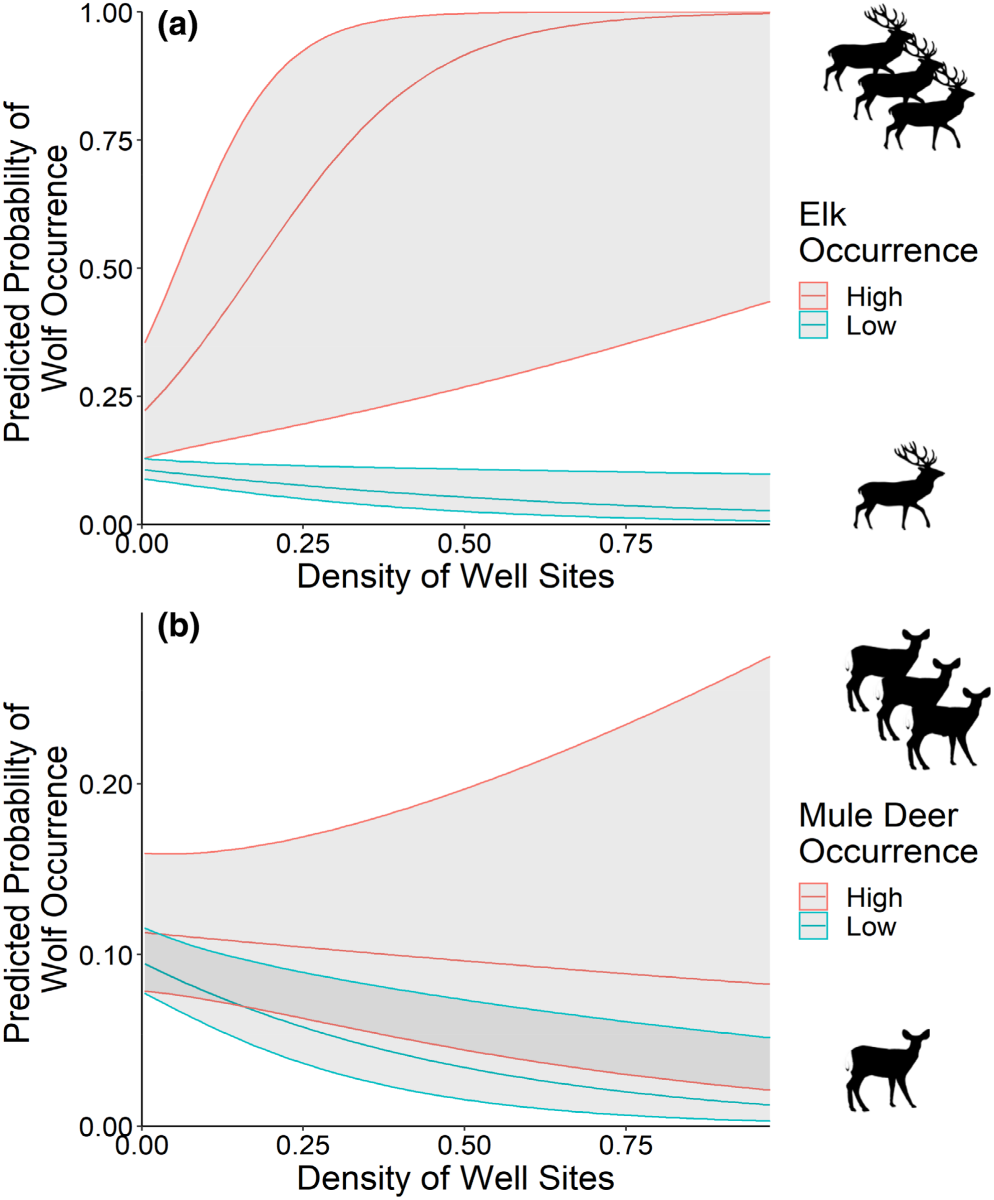
Relative probability of wolf (*Canis lupus*) occurrence, with 95% confidence intervals, as a function of the density of industrial well sites in the presence of high (95th percentile; red) and low (5th percentile; blue) occurrence frequencies of (a) elk (*Cervus canadensis*) and (b) mule deer (*Odocoileus hemionus*), based on camera trap data collected in central Alberta's Rocky Mountains and foothills regions during 2014.

**FIGURE 6:**
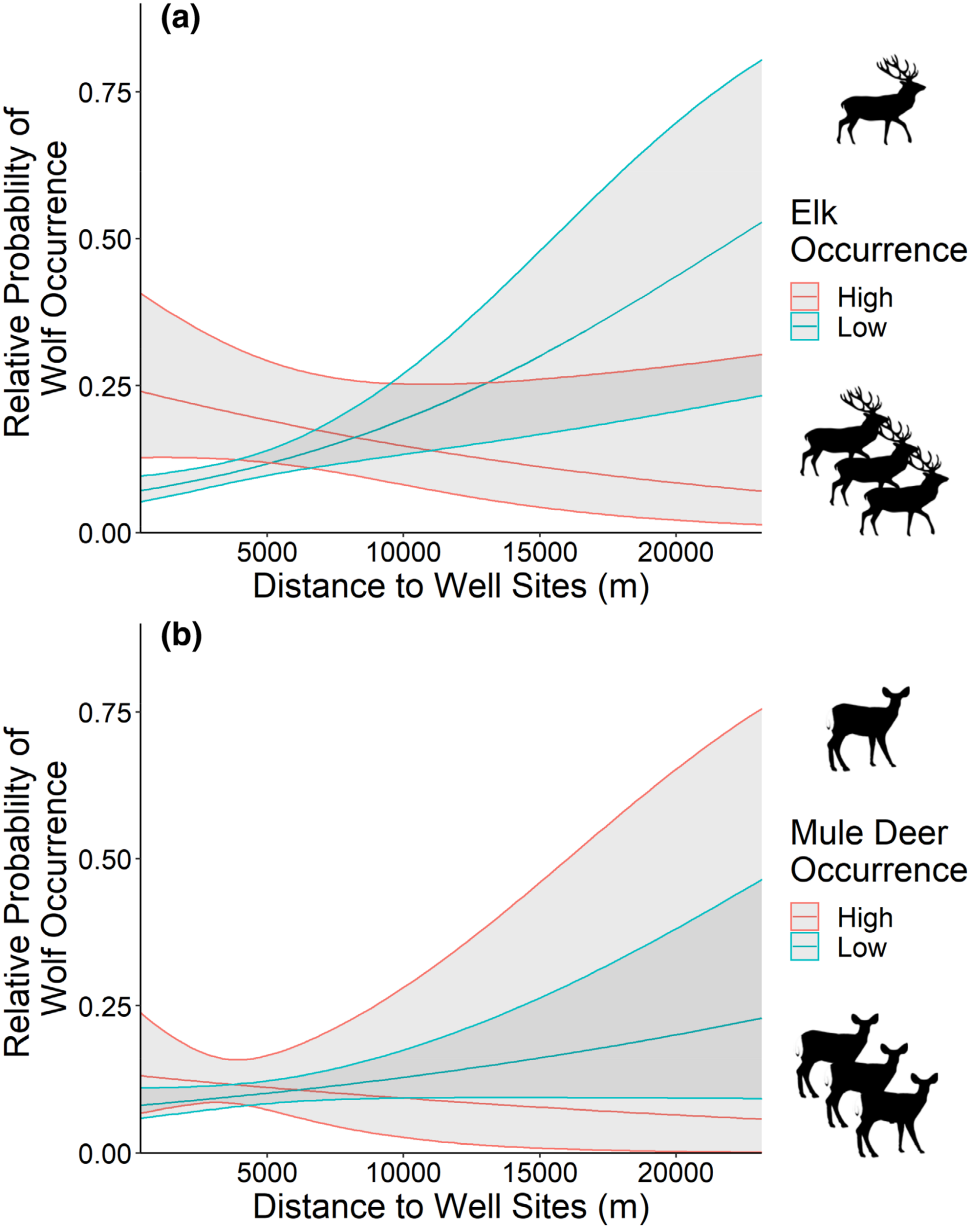
Relative probability of wolf (*Canis lupus*) occurrence, with 95% confidence intervals, as a function of the distance to industrial well sites in the presence of high (95th percentile; red) and low (5th percentile; blue) occurrence frequencies of (a) elk (*Cervus canadensis*) and (b) mule deer (*Odocoileus hemionus*), based on camera trap data collected in central Alberta's Rocky Mountains and foothills regions during 2014.

**FIGURE 7:**
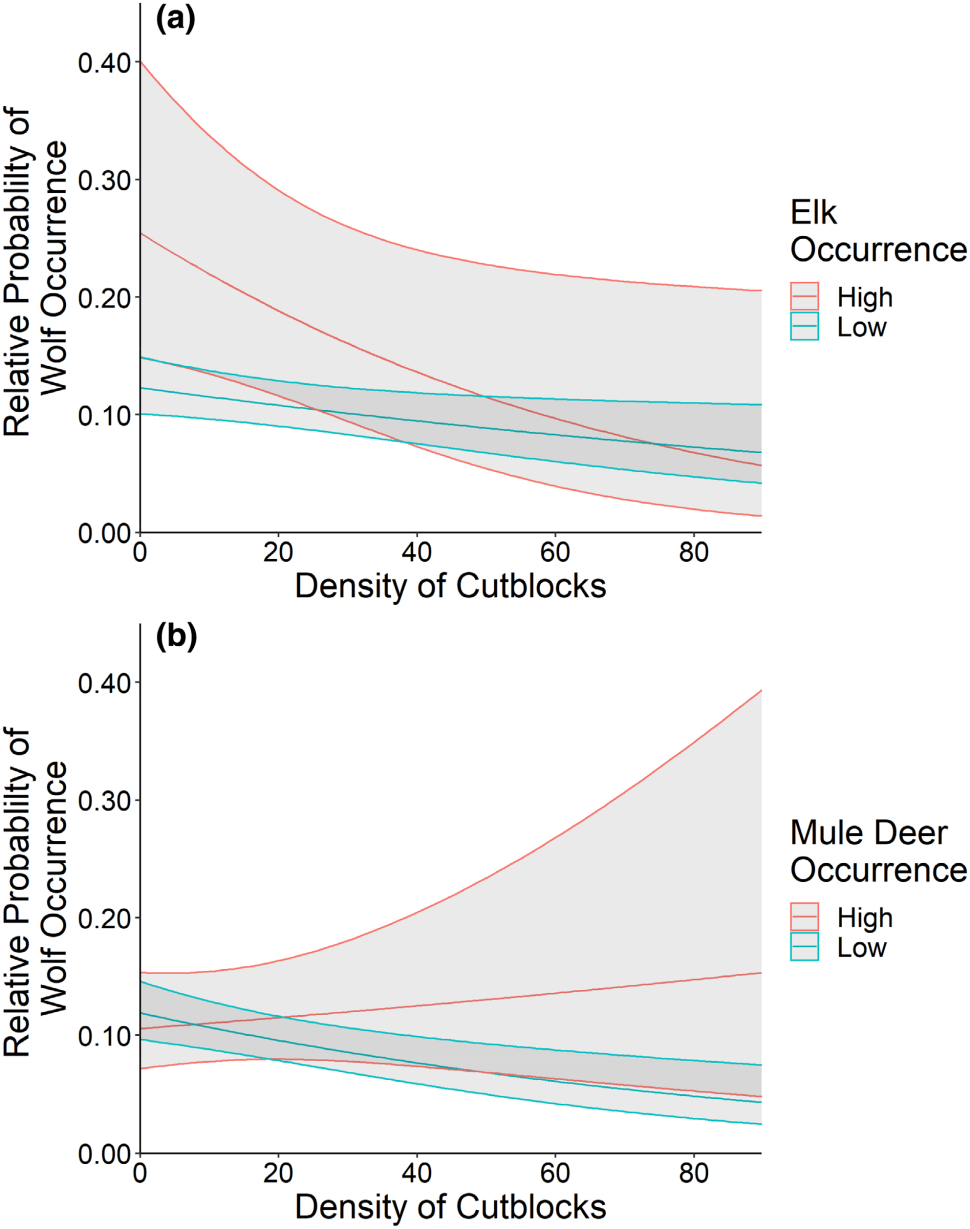
Relative probability of wolf (*Canis lupus*) occurrence, with 95% confidence intervals, as a function of the density of cutblocks in the presence of high (95th percentile; red) and low (5th percentile; blue) occurrence frequencies of (a) elk (*Cervus canadensis*) and (b) mule deer (*Odocoileus hemionus*), based on camera trap data collected in central Alberta's Rocky Mountains and foothills regions during 2014.

Sites where elk occurred frequently on industrial block features were the strongest predictors of wolf occurrences (Table 2). Wolves occurred more frequently with increasing occurrences of elk (*β* ± SE = 0.27 ± 0.13) and distances farther from well sites (*β* ± SE = 0.36 ± 0.12) (Figure 4a). Wolves occurred less frequently in areas with high cutblock densities (*β* ± SE = −0.24 ± 0.08), but we observed no trend in relation to well site densities (*β* ± SE = 0.02 ± 0.13). These relationships were influenced by the interaction between block features and the frequency of elk occurrence, as wolf occurrence frequency increased with higher density of well sites (*β* ± SE = 0.75 ± 0.27) and proximity to well sites (*β* ± SE = −0.30 ± 0.11) when sites had more frequent occurrences of elk (Figures 5a and 6a). However, wolves still showed decreased occurrences at high cutblock densities when elk were detected frequently (*β* ± SE = −0.12 ± 0.10) (Figure 7a).

### 3.4 Wolf occurrence relative to mule deer and block features

Wolves occurred more frequently at sites where mule deer occurred frequently, in combination with industrial block features (AIC_w_ = 0.37, Table 2, Figure 4b). Occurrences of wolves decreased at high well site densities (*β* ± SE = −0.37 ± 0.11), closer to well sites (*β* ± SE = 0.13 ± 0.11), and at high cutblock densities (*β* ± SE = −0.20 ± 0.07) (Figure 4b). Wolf occurrences showed no significant trend in relation to mule deer occurrence frequency alone (*β* ± SE = 0.06 ± 0.09). However, when mule deer were detected frequently, wolf occurrences increased in habitats with higher well site (*β* ± SE = 0.16 ± 0.07) and cutblock densities (*β* ± SE = 0.17 ± 0.09) (Figures 5, 6, 7).

### 3.5 Wolf occurrence relative to natural land cover

Wolf occurrences were positively related to the proportion of open wetland habitat (*β* ± SE = 0.30 ± 0.07) and water features (*β* ± SE = 0.15 ± 0.05) on the landscape (Figure 4a). However, wolf occurrence decreased at higher elevations (*β* ± SE = −0.49 ± 0.10) and with increasing proportions of barren land (*β* ± SE = −1.07 ± 0.17), dense conifer (*β* ± SE = −0.28 ± 0.08), and mixed wood (*β* ± SE = −0.39 ± 0.10). Beta estimates are reported from the elk model but were similar between both supported models (Figure 4).

## 4 DISCUSSION

The frequency of wolf occurrences in relation to industrial block features (well sites and cutblocks) was mediated by elk and mule deer availability, suggesting a complex relationship between predators, prey, and anthropogenic features exists in landscapes with extensive resource extraction. When prey were detected infrequently, wolves occurred less at sites with high densities of cutblocks and well sites, as well as habitat near well sites. However, when elk or mule deer occurred frequently on these anthropogenic features, so did wolves. Based on these results, wolves risk using these industrial block features to increase predation opportunities when prey occur frequently, but otherwise occur less on them due to the perceived risk from humans.

### 4.1 Wolf avoidance of industrial block features

We identified well sites and cutblocks as important drivers of wolf distributions within this system. Previous research has found that these industrial block features influence wolf habitat selection, although with varying responses (Boucher et al., 2022; Houle et al., 2010; Kittle et al., 2017; Muhly et al., 2019; Spilker, 2019). Hebblewhite (2006) found positive selection for cutblocks by wolves, whereas our models indicated that wolves occur less in areas with cutblocks, a similar result to Spilker (2019). Wolf use of cutblocks could vary with different levels of logging activity because wolves often occur less frequently in areas with a high probability of human encounters (Rogala et al., 2011; Whittington et al., 2005). Our results also show evidence of wolves reducing their presence around well sites, possibly due to the perceived risk of humans encounters on these features (Rogala et al., 2011; Whittington et al., 2005). This relationship of avoidance of industrial features due to human activity has been documented in previous work, where wolves adopt strategies to minimize contact with humans, including spatial avoidance (Khan et al., 2023; Muhly et al., 2019; Spilker, 2019; Theuerkauf, 2009). Industrial features, such as cutblocks, are often associated with an increased mortality risk for wolves due to hunting or trapping by humans (Person & Russell, 2008). Wolf harvest accounts for most adult mortalities in Alberta's foothills (Hebblewhite & Whittington, 2020; Webb et al., 2011), possibly elevating wolves' risk around anthropogenic features. Conversely, our models including motorized and non‐motorized human activity were not strongly supported, indicating that presence of industrial block features plays a more significant role in wolf occurrence than human activity itself. Possibly, wolves perceive these block features as risky places, regardless of human activity. This is supported by low wolf occurrences around well sites, despite a high proportion of these features being abandoned. However, we did not quantify the risk from humans (e.g., hunting) in relation to motorized and non‐motorized activity. As well, we did not explore the temporal aspects of wolf occurrence frequency thus we could not examine whether wolves were temporally but not spatially avoiding high human activity. We also did not examine age since disturbance, which could influence human activity on these features as well as wolf occurrence. Additionally, our measure of human activity was localized at the camera site and may not be fully representative of active industrial work or recreational activity within the area. Other mechanisms that could lead to wolf avoidance of these anthropogenic features, such as altered hunting success, should also be explored in future studies.

### 4.2 Prey availability mediates wolf occurrences on industrial block features

While wolves spatially separated from block features, this relationship was modified by prey availability. Wolves occurred more frequently in habitats with block features that had higher occurrences of either elk or mule deer. Ungulates are known to select regenerating disturbance patches, such as cutblocks and well sites, due to increased available early seral forage (Bowman et al., 2010; Fisher & Burton, 2018; Latham, Latham, McCutchen, et al., 2011; Muhly et al., 2011, 2019). Increased selection of industrial block features by elk or mule deer would increase predation opportunities for wolves on these features, altering the risk versus reward payoffs for wolves using these habitats. Future studies could improve upon our results by modeling prey habitat selection in relation to block features, resource availability, and predation risk.

Models including other prey species (moose and white‐tailed deer) in relation to block features were not as well supported, which is consistent with previous findings that mule deer and elk are preferred prey for wolves in the Rocky Mountains and foothills regions (Huggard, 1993; MacAulay, 2019; Wasser et al., 2011). Moose occur at a low frequency in wolf diets in Alberta's foothills (MacAulay, 2019; MacAulay et al., 2022), but white‐tailed deer are often a selected prey species. While it is possible that white‐tailed deer do not significantly mediate wolf occurrence on anthropogenic features, the relatively ubiquitous distribution of white‐tailed deer across the study area could have resulted in a limited ability to detect their influence on wolf occurrences. Additionally, white‐tailed deer may play a stronger role on wolf occurrence in respect to natural features, but we only explored how prey availability influenced occurrence on anthropogenic features. As well, we did not examine the relationship of wolf occurrence frequency to other predators (e.g., grizzly bears, *Ursus arctos*), ungulate species with limited detections (e.g., bighorn sheep and caribou), or non‐ungulate prey species (e.g., snowshoe hare, *Lepus americanus*) present in this landscape, but these other species likely also influence wolf habitat use and should be included in future research.

### 4.3 Wolf response to linear features

Unexpectedly, we did not find strong support for models relating wolf occurrence frequency to linear features. Our results differ from previous studies, which find wolf selection of linear features for improved search efficiency during hunting via increased movement rates (Boucher et al., 2022; DeMars & Boutin, 2018; Dickie et al., 2017). Furthermore, spatial overlap of wolves and ungulate refugia is facilitated by linear features, resulting in an increased wolves' ability to reach vulnerable species such as caribou (Dickie et al., 2017; Latham, Latham, Boyce, et al., 2011). Conversely, linear features also create refugia for ungulates, due to wolf avoidance of humans (Muhly et al., 2011).

Our models did not provide strong evidence that linear features influenced wolf occurrence frequency on this landscape, but this does not mean this relationship is not present. Possibly, high prey densities in our study area made selection by wolves for potentially prey‐rich habitats (e.g., cutblocks) more optimal than selecting for linear features which improve hunting efficiency at low prey densities (Kittle et al., 2017). Additionally, the relationship between wolves and linear features may not be well identified within our study because all cameras were placed on human‐created trails. Our study design might impede our ability to fully assess the influence of linear features, and species' detections also could be influenced by these camera site locations. However, this study design was required to assess motorized and non‐motorized human activity and we distributed camera sites across varying linear feature densities to account for camera placement on human‐use trails.

### 4.4 Trade‐offs by wolves for predation opportunities and risk from humans

Our most substantially supported candidate models suggest that the frequency of wolf occurrence changed when elk or mule deer are abundant on industrial block features. When prey were not detected frequently on block features, wolf occurrence frequency decreased; however, wolf occurrence on the landscape shifted when prey availability increased. In areas with abundant mule deer or elk, wolves occurred frequently at high well site densities, closer to well sites, and high cutblock densities. Unlike with elk, wolves did not exhibit evident co‐occurrence with mule deer when the interaction with block features was not considered, suggesting that the abundance of mule deer did not determine wolf presence unless they were present on block features. As these features supported higher densities of prey species likely due to available early seral forage, we can predict that wolves selected these features for increased predation opportunities (Fisher & Burton, 2018; Kittle et al., 2017; Latham, Latham, McCutchen, et al., 2011; Muhly et al., 2019). Likely, wolves occur on these block features only when hunting opportunities are present, because these areas would offer little incentive if prey abundance is low but human encounter risk is high. Wolves often select for high prey densities to improve hunting success and are also documented to select well sites for hunting, even though they may be less likely to kill on these features due to human disturbances (McPhee et al., 2012). As well, ungulate kill sites are found to increase at higher proportions of cutblocks, in combination with wolf selection for young cutblocks (Boucher et al., 2022).

Wolves make foraging decisions based on the prevalent landscape conditions and prey availability (Kittle et al., 2017; Muhly et al., 2019; Theuerkauf, 2009). Our results align with conclusions of Kittle et al. (2017), who found that wolves selected disturbed non‐treed habitat to improve hunting success at high prey densities but will prioritize use of linear features for mobility at low prey densities. We found that wolves occurred less frequently on industrial block features unless prey were present and that human activity was not well supported as a predictor of wolf occurrences. We suggest that wolves exhibit a plastic response to industrial block features, making trade‐offs between risk avoidance and hunting opportunities based on prey availability. When prey availability is low on industrial block features, wolves likely prioritize avoidance of risk from humans and exhibit spatial separation from these features. Conversely, when prey occur more frequently on industrial block features, hunting opportunities for wolves increase such that wolves will use these risky features. Wolves appear to prioritize factors such as prey availability, anthropogenic features, and the perceived risks of human presence, rather than recreational human activity itself, when making foraging decisions.

## 5 CONCLUSIONS

In boreal mountain ecosystems, wolf spatial distribution is a complex response to prey availability and industrial block features, such that industrial development likely leads to shifts in predator–prey dynamics in this system. We identified that industrial block features negatively influence wolf occurrence frequency on the landscape, but this response is reversed when predation opportunities are present. Block features appear to only be perceived as beneficial by wolves when elk or mule deer frequently occur on these features, indicating that wolves trade‐off between risk from humans and hunting opportunities, depending on prey availability. Our results provide evidence for the importance of cohesively considering multiple species when modeling predator distributions, because the interaction between prey availability and industrial block features had a greater influence on wolf occurrence frequency than when these variables were considered alone. Effective management of wolf habitats and populations in human‐altered landscapes thus requires the simultaneous consideration of block features and these prey species. Overall, our results indicate that industrial block features are changing predator–prey relationships in human‐altered landscapes by altering wolf distributions and the spatial co‐occurrence between wolves and their prey, which could lead to broad‐scale implications for predator and prey populations.

## AUTHOR CONTRIBUTIONS

**Hannah Boczulak:** Conceptualization (equal); formal analysis (equal); investigation (equal); methodology (equal); validation (equal); visualization (equal); writing – original draft (equal); writing – review and editing (equal). **Nicole P. Boucher:** Formal analysis (equal); investigation (equal); methodology (equal); validation (equal); visualization (equal); writing – review and editing (equal). **Andrew Ladle:** Conceptualization (equal); formal analysis (equal); funding acquisition (equal); investigation (equal); methodology (equal); supervision (equal). **Mark S. Boyce:** Conceptualization (equal); data curation (equal); funding acquisition (equal); investigation (equal); methodology (equal); project administration (equal); supervision (equal); writing – review and editing (equal). **Jason T. Fisher:** Conceptualization (equal); data curation (equal); funding acquisition (equal); investigation (equal); methodology (equal); project administration (equal); supervision (equal); writing – review and editing (equal).

## CONFLICT OF INTEREST STATEMENT

The authors declare that there are no conflicts of interest.

## Data Availability

Data and associated R scripts are available at https://doi.org/10.5683/SP3/3PRPIF.
